# Positive relationship between Work-to-Sleep hours Ratio and obesity: a cross-sectional study, evidence from NHANES 2017–2023

**DOI:** 10.3389/fpubh.2025.1616890

**Published:** 2025-06-16

**Authors:** Jinggang Zhou

**Affiliations:** Department of Gastroenterology, Jiangyou People’s Hospital, Jiangyou, China

**Keywords:** Work-to-Sleep hours Ratio, obesity, work hours, sleep hours, NHANES

## Abstract

**Background:**

Current approaches relying solely on work hours or sleep hours often fall short in comprehensively assessing health risks. To address this gap, this study introduces a novel metric: the Work-to-Sleep hours Ratio (WSR). The study aims to investigate the relationship between WSR and obesity.

**Objective:**

To investigate the correlation between WSR and obesity.

**Methods:**

We employing data from 7,847 participants in the National Health and Nutrition Examination Survey (NHANES) 2017–2023. Data collected from all participants included demographic variables, health-related metrics and the presence of various health conditions. Logistic regression analysis, Restricted Cubic Spline (RCS) analysis, and interaction effects were employed to support the research objectives.

**Results:**

In the final model of multivariate analysis showed positive relationship between WSR and obesity (OR = 1.54, 95% CI:1.33–1.77, *p* < 0.001). Additionally, multivariate smooth splines analysis indicated that WSR exhibited a significant inverted L-shaped nonlinear relationship with obesity (P for nonlinearity < 0.05).

**Conclusion:**

The study observed a positive correlation between WSR and obesity, highlighting the importance of considering both work and sleep hours in assessing public health risks.

## Introduction

Obesity has become a severe global health issue. As World Health Organization (WHO) criteria, individuals with body mass index (BMI) ≥ 30 kg/m^2^ are considered obesity (1). WHO data shows that the global obesity population reached 890 million in 2022 (1) with this number projected to grow to 1.12 billion by 2030 (2). By 2030, almost half of American adults will grapple with obesity. Nationwide, no state will have an obesity prevalence rate lower than 35% and the rate will exceed 50% in 29 states (3). Severe obesity will afflict nearly one - fourth of women, non - Hispanic Black adults, and low-income adults (3). Obesity is a high-risk factor for numerous diseases (4, 5). In terms of the cardiovascular system (5–8), a large number of studies have confirmed that obesity can significantly increase the risk of coronary heart disease (CHD), myocardial infarction, and cerebral infarction. Obesity enhances insulin resistance (7, 9) and increases the likelihood of diabetes (6). In addition, obesity lead to obstructive sleep apnea syndrome (10), which affects the quality of sleep and causes damage to vital organs (10) such as the heart, brain, and kidneys. Moreover, obesity is closely related to the occurrence and development of diseases such as cancer (9) and osteoarthritis (11).

Factors contributing to obesity can be categorized into sociodemographic, behavioral, genetic, and living in obesogenic environment (12, 13). Researches hold that prolonged work hours (14–16) and short sleep duration (17–19) have become important factors contributing to obesity. Currently, the predominant work schedule is five workdays a week, with 8 h of work per workday. However, extended work hours are becoming increasingly common in many parts of the world. According to the information released by the International Labour Organization (ILO) (20), prior to the COVID-19 pandemic, the average number of weekly work hours for paid employment globally was approximately 43.9 h. Notably, around one-third (35.4%) of the workforce worked more than 48 h per week (20). In Japan (21) approximately 25% of Japanese companies reported that their employees, on average, worked overtime for more than 80 h per month, and a significant amount of this overtime work was not recorded (22). In South Korea, the standard work hours per week are 52 h (20). Long work hours badly invade employees’ personal lives, restricting daily activities and disrupting sleep patterns.

Existing studies have examined the relationship between work hours (15, 23) or sleep hours (19, 24) and obesity. However, there is still a shortage of research that comprehensively takes into account both work and sleep hours. Therefore, we propose a brand-new indicator, the WSR, to make up for this deficiency. In this study, both work and sleep hours are measured on a weekly basis. This paper addresses this gap through a cross-sectional study, which included 7,847 participants with aged ≥ 18 years. The value of WSR is equal to the value of work hours divided by sleep hours (WSR = work hours/sleep hours).

## Materials

### Participants

Participants were recruited from the NHANES 2017–2023. The ethical review and informed consent procedures for the NHANES study have been approved by the National Center for Health Statistics (NCHS) Institutional Review Board.^1^ Therefore, this dataset does not require additional ethical review. This study adhered to the Strengthening the Reporting of Observational Studies in Epidemiology (STROBE) reporting guidelines.

### Inclusion and exclusion

**Figure 1:**
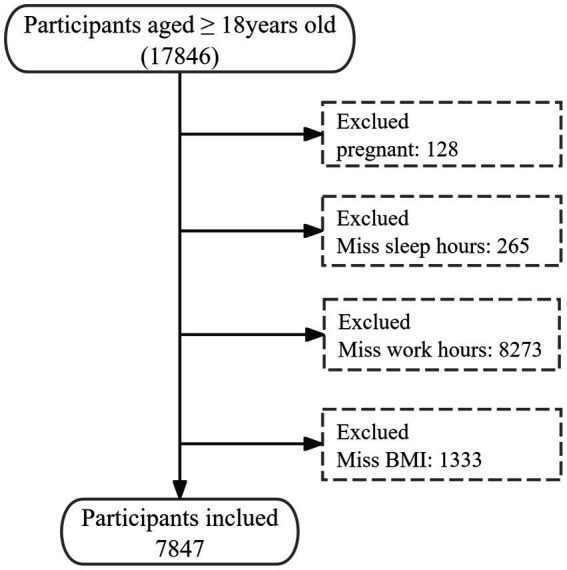
Flow diagram of the sample selection.

Data screening was conducted using R, version 4.4.4 (R Core Team, 2023). Missing BMI values were imputed using statistical software from the Free Software Foundation (version 2.1; Beijing Free Clinical Medical Technology Co., Ltd.) (25). The process of data inclusion and exclusion is illustrated in Figure 1. The data will be utilized to facilitate the implementation of various other research.

### Covariates

In this study, covariates were selected based on a review of similar literature including age, gender, race, marital status, education level, BMI, poverty income ratio (PIR), alcohol consumption status, smoking status, time of vigorous and moderate leisure-time physical activity (VLTPA and MLTPA), sedentary activity, energy (kcal), heart failure (HF), CHD, diabetes mellitus (DM), hypertension, hyperlipidemia, stroke, chronic obstructive pulmonary disease (COPD), thyroid problem, cancer, arthritis. The quantification of MLTPA, VLTPA and sedentary activity is based on daily minute counts.

#### Work hours, sleep hours, WSR and obesity

In this study, Work hours come from the response to the question “Number of hours worked in the last week.”Sleep hours were calculated by multiplying the response to the question “Sleep hours - weekdays or workdays” by 5 and the response to “Sleep hours - weekends” by 2, then summing the two products [Sleep hours = (weekday sleep × 5) + (weekend sleep × 2)]. WSR = work hours/sleep hours. BMI ≥ 30 was defined as obesity.

#### Other covariates

In this study, Individuals with age ≥60 year were classified as elder. PIR less than 1 were classified as poor. Values of MLTPA, VLTPA, sedentary activity, energy are derived from the answers to the questionnaire.

For race, the groups “Other Hispanic” and “Other Race - Including Multi-Racial” were combined into a single category labeled “Other Races,” while all other racial categories were retained as per the questionnaire. Participants who had completed more than the 11th grade were categorized as “High School or above.” Individuals who answered “Married/Living with partner” or “Widowed/Divorced/Separated” were classified as married. Alcohol consumption status was assessed based on responses to the questions “Ever had a drink of any kind of alcohol” and “Past 12 mos how often drink alc bev.” Individuals who answered “Yes” to the first question and reported more than zero days of alcohol consumption in the past 12 months were identified as drinkers. Individuals responses to the questions “Have you smoked at least 100 cigarettes in your lifetime?” and “Do you now smoke cigarettes?” Participants who answered “Yes” to both questions were classified as smokers.

The presence of HF, CHD, DM, hypertension, hyperlipidemia, stroke, COPD, thyroid problem, cancer, and arthritis was determined based on affirmative (“Yes”) responses to the corresponding questions in the questionnaire, such as “Have you/Has SP ever been told by a doctor or other health professional that you/s/he had cancer or a malignancy (ma-lig-nan-see) of any kind?”

### Statistical analyses

Data analysis were performed using R, version4.4.1, along with Zstats v1.0^2^ and Free Software Foundation statistics software (version 2.1; Beijing Free Clinical Medical Technology Co., Ltd.) (25). For skewed continuous data, they are presented as M (Q₁, Q₃), and the Mann–Whitney U test is used for comparison. For normally distributed continuous data, they are expressed as Mean ± SD, and the independent samples *t*-test is applied for comparison between two groups. Categorical data are presented as *n* (%), and the chi-square test or Fisher’s exact test is used. A *p*-value < 0.05 is considered statistically significant for intergroup comparisons.

We employed a multivariate logistic regression model to examine the association between WSR and obesity. Four models were constructed for this analysis. The last model was adjusted for age, gender, race, marital status, education level, BMI, PIR, alcohol consumption status, smoking status, MLTPA, VLTPA, sedentary, energy, HF, CHD, DM, hypertension, hyperlipidemia, stroke, COPD, thyroid problem, cancer, arthritis.

## Results

### Baseline characteristics of the population

Table 1 presents the basic characteristics of 7,847 individuals drawn from the NHANES data rounds of 2017–2020 and 2021–2023. Among these participants, approximately 49.94% (3,196/7847) exhibited obesity. The mean age of the study population was 44.11 ± 15.31 years, with 3,855(49.13%) participants being female.

**Table 1:** Characteristics of participants.

Variables	Total (*n* = 7,847)	Non-Obesity (*n* = 4,651)	Obesity (*n* = 3,196)	Statistic	*p*
Age, Mean ± SD	44.11 ± 15.31	43.42 ± 15.74	45.12 ± 14.61	*t* = −4.90	<0.001
BMI (kg/m^2^), Mean ± SD	29.69 ± 7.42	24.93 ± 3.13	36.62 ± 6.30	*t* = −96.94	<0.001
Work hours, Mean ± SD	39.13 ± 14.01	38.41 ± 14.05	40.17 ± 13.90	*t* = −5.50	<0.001
Sleep hours, Mean ± SD	53.61 ± 9.06	54.01 ± 9.00	53.03 ± 9.11	*t* = 4.70	<0.001
WSR, M (Q₁, Q₃)	0.74 (0.58, 0.91)	0.73 (0.56, 0.90)	0.76 (0.62, 0.94)	*Z* = −6.21	<0.001
Energy (Kcal), M (Q₁, Q₃)	2000.00 (1537.50, 2524.75)	2009.50 (1545.25, 2520.75)	1969.25 (1520.38, 2526.50)	*Z* = −1.44	0.149
VLTPA, M (Q₁, Q₃)	60.00 (30.00, 75.00)	60.00 (30.00, 85.50)	60.00 (30.00, 60.00)	*Z* = −0.05	0.962
MLTPA, M (Q₁, Q₃)	60.00 (30.00, 60.00)	60.00 (30.00, 60.00)	60.00 (30.00, 60.00)	*Z* = −0.04	0.969
Sedentary activity, M (Q₁, Q₃)	300.00 (180.00, 480.00)	300.00 (180.00, 480.00)	360.00 (180.00, 540.00)	Z = −7.30	<0.001
Gender, *n* (%)				*χ*^2^ = 25.51	<0.001
Male	3,992 (50.87)	2,476 (53.24)	1,516 (47.43)		
Female	3,855 (49.13)	2,175 (46.76)	1,680 (52.57)		
Education, *n* (%)				*χ*^2^ = 0.09	0.763
Below high school	867 (11.05)	518 (11.14)	349 (10.92)		
High School or above	6,980 (88.95)	4,133 (88.86)	2,847 (89.08)		
Race, *n* (%)				*χ*^2^ = 179.79	<0.001
Mexican American	870 (11.09)	454 (9.76)	416 (13.02)		
Non-Hispanic White	3,258 (41.52)	1912 (41.11)	1,346 (42.12)		
Non-Hispanic Black	1,620 (20.64)	811 (17.44)	809 (25.31)		
Other	2099 (26.75)	1,474 (31.69)	625 (19.56)		
Marital, *n* (%)				*χ*^2^ = 5.77	0.016
No	2065 (26.32)	1,270 (27.31)	795 (24.87)		
Yes	5,782 (73.68)	3,381 (72.69)	2,401 (75.13)		
PIR, *n* (%)				*χ*^2^ = 1.89	0.170
≥ 1	6,991 (89.09)	4,125 (88.69)	2,866 (89.67)		
< 1	856 (10.91)	526 (11.31)	330 (10.33)		
Smoker, *n* (%)				*χ*^2^ = 7.56	0.006
Yes	1,211 (15.43)	761 (16.36)	450 (14.08)		
No	6,636 (84.57)	3,890 (83.64)	2,746 (85.92)		
Drinker, *n* (%)				*χ*^2^ = 0.82	0.366
Yes	5,769 (73.52)	3,402 (73.15)	2,367 (74.06)		
No	2078 (26.48)	1,249 (26.85)	829 (25.94)		
DM, *n* (%)				*χ*^2^ = 137.46	<0.001
Yes	675 (8.60)	257 (5.53)	418 (13.08)		
No	7,172 (91.40)	4,394 (94.47)	2,778 (86.92)		
Hypertension, *n* (%)				*χ*^2^ = 286.20	<0.001
Yes	2095 (26.70)	916 (19.69)	1,179 (36.89)		
No	5,752 (73.30)	3,735 (80.31)	2017 (63.11)		
Hyperlipidemia, *n* (%)				*χ*^2^ = 69.32	<0.001
Yes	2,261 (28.81)	1,176 (25.28)	1,085 (33.95)		
No	5,586 (71.19)	3,475 (74.72)	2,111 (66.05)		
Arthritis, *n* (%)				*χ*^2^ = 102.24	<0.001
Yes	1,458 (18.58)	693 (14.90)	765 (23.94)		
No	6,389 (81.42)	3,958 (85.10)	2,431 (76.06)		
HF, *n* (%)				*χ*^2^ = 20.69	<0.001
Yes	104 (1.33)	39 (0.84)	65 (2.03)		
No	7,743 (98.67)	4,612 (99.16)	3,131 (97.97)		
CHD, *n* (%)				*χ*^2^ = 1.13	0.287
Yes	156 (1.99)	86 (1.85)	70 (2.19)		
No	7,691 (98.01)	4,565 (98.15)	3,126 (97.81)		
Stroke, *n* (%)				*χ*^2^ = 2.85	0.091
Yes	127 (1.62)	66 (1.42)	61 (1.91)		
No	7,720 (98.38)	4,585 (98.58)	3,135 (98.09)		
COPD, *n* (%)				*χ*^2^ = 17.25	<0.001
Yes	296 (3.77)	141 (3.03)	155 (4.85)		
No	7,551 (96.23)	4,510 (96.97)	3,041 (95.15)		
Thyroid problem, *n* (%)				*χ*^2^ = 16.54	<0.001
Yes	642 (8.18)	332 (7.14)	310 (9.70)		
No	7,205 (91.82)	4,319 (92.86)	2,886 (90.30)		
Cancer, *n* (%)				χ^2^ = 0.21	0.650
Yes	511 (6.51)	298 (6.41)	213 (6.66)		
No	7,336 (93.49)	4,353 (93.59)	2,983 (93.34)		
Age, *n* (%)				*χ*^2^ = 1.57	0.210
< 60	6,291 (80.17)	3,707 (79.70)	2,584 (80.85)		
≥ 60	1,556 (19.83)	944 (20.30)	612 (19.15)		
Sleep duration (hours), *n* (%)				*χ*^2^ = 22.62	<0.001
< 39	2,511 (32.00)	1,404 (30.19)	1,107 (34.64)		
≥ 39, < 75	2,637 (33.61)	1,563 (33.61)	1,074 (33.60)		
≥ 75	2,699 (34.40)	1,684 (36.21)	1,015 (31.76)		

*t*, *t*-test; Z, Mann–Whitney test; χ^2^, Chi-square test; SD, standard deviation; M, Median; Q₁, 1st Quartile; Q₃, 3st Quartile. Energy, Total energy intake, Kcal, Work and sleep hours: Hours/week, VLTPA, MLTPA, Sedentary activity: Minutes/day.

Table 1 reveals that 3,196 individuals with obesity have a higher proportion of females, married individuals, non-smokers, DM, hypertension, hyperlipidemia, angina, HF, thyroid problems, and COPD. Additionally, this group tends to be older, longer work hours, have shorter sleep duration, higher WSR and sedentary activity. In Table 1, individuals with obesity have a higher proportion of individuals with sleep hours < 39 h/week, and lower proportion of sleep hours ≥ 75 h/week (*χ*^2^ = 22.62, *p* < 0.001).

### The correlation between WSR and obesity

**Figure 2:**
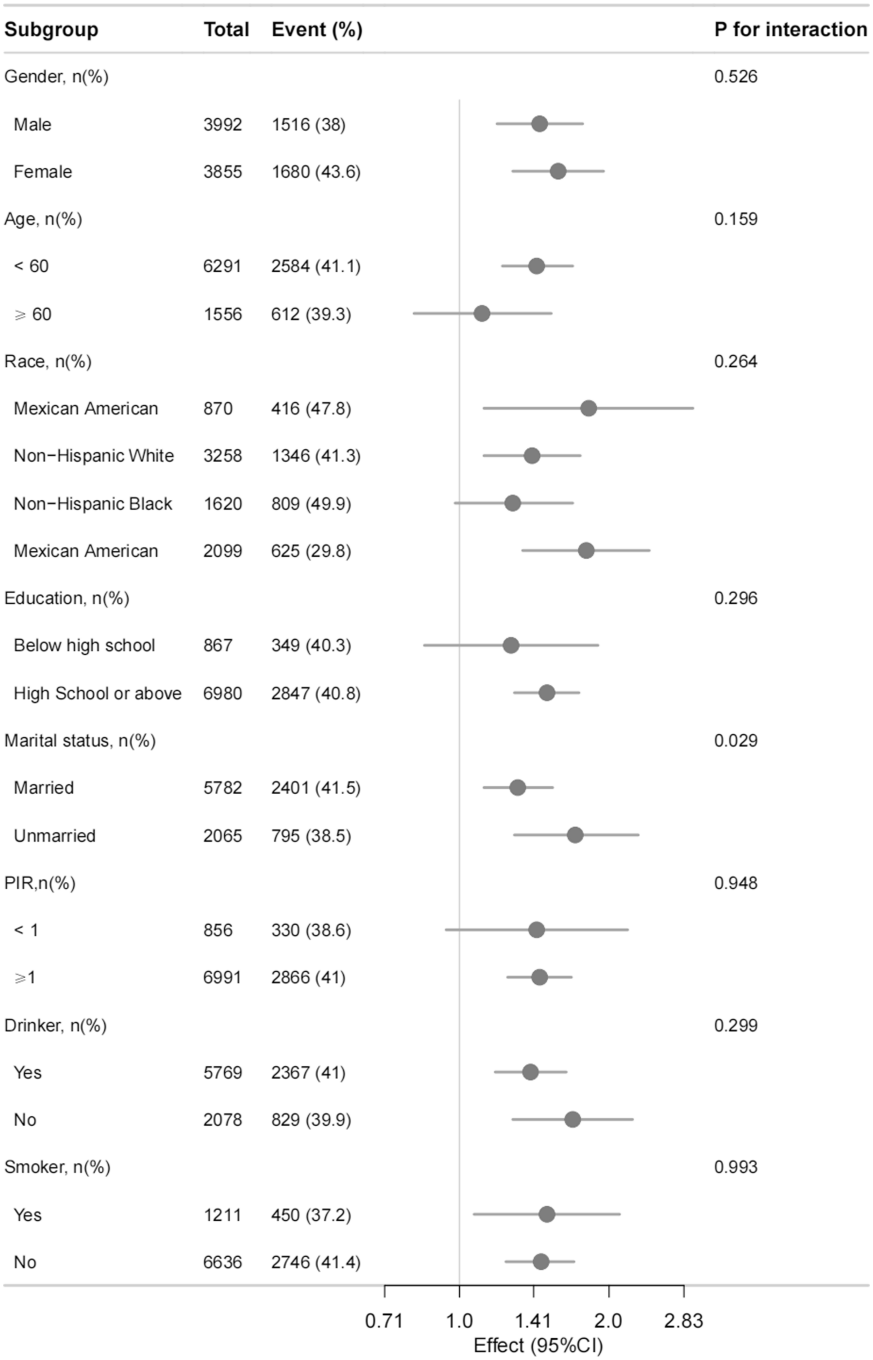
Association between WSR and obesity in different subgroups. Adjusted for othertime, age, gender, race, marital status, education level, BMI, PIR, alcohol consumption, smoking status, MLTPA, VLTPA, sedentary activity, HF, CHD, DM, hypertension, hyperlipidemia, stroke, COPD, thyroid problem, cancer and arthritis.

A significant association was observed between WSR and obesity in the univariate analyses (OR = 1.49, 95% CI = 1.31–1.70, *p* < 0.001, Supplementary Table 1). In the final model of multivariate analysis, WSR was identified as an independent risk factor for obesity. Specifically, compared with work hours (OR = 1.01, 95% CI = 1.01–1.01, *p* < 0.001) or sleep hours (OR = 0.99, 95% CI 0.98–0.99, *p* < 0.001) alone, WSR had a more pronounced effect on obesity (OR = 1.54, 95% CI = 1.33–1.77, *p* < 0.001) (Table 2). Subgroup analyses were conducted and no significant interactions was observed (P for interaction > 0.05) (Figure 2).

**Table 2:** Multivariate regression analysis of the association between WSR/work hours/sleep duration and obesity.

Variables	Mode I		Mode II		Mode III		Mode IV	
	adj. OR (95%CI)	*p* value	adj. OR (95%CI)	*p* value	adj. OR (95%CI)	*p* value	adj. OR (95%CI)	*p* value
WSR	1.57 (1.38 ~ 1.8)	<0.001	1.56 (1.36 ~ 1.79)	<0.001	1.57 (1.37 ~ 1.8)	<0.001	1.54 (1.33 ~ 1.77)	<0.001
Work hours	1.01 (1.01 ~ 1.01)	<0.001	1.01 (1.01 ~ 1.01)	<0.001	1.01 (1.01 ~ 1.01)	<0.001	1.01 (1.01 ~ 1.01)	<0.001
Sleep hours	0.99 (0.98 ~ 0.99)	<0.001	0.99 (0.98 ~ 0.99)	<0.001	0.99 (0.98 ~ 0.99)	<0.001	0.99 (0.98 ~ 0.99)	<0.001
Sleep duration (hours)								
≥ 39, < 75	1(Ref)		1(Ref)		1(Ref)		1(Ref)	
< 39	1.15 (1.03 ~ 1.29)	0.012	1.13 (1.01 ~ 1.27)	0.03	1.14 (1.02 ~ 1.28)	0.022	1.12 (1 ~ 1.26)	0.056
≥ 75	0.88 (0.79 ~ 0.98)	0.022	0.87 (0.77 ~ 0.97)	0.013	0.87 (0.78 ~ 0.98)	0.017	0.86 (0.77 ~ 0.97)	0.011
Trend.test		<0.001		<0.001		<0.001		<0.001
WSR < 1.205								
WSR	1.86 (1.53 ~ 2.26)	<0.001	1.91 (1.56 ~ 2.33)	<0.001	1.89 (1.55 ~ 2.31)	<0.001	1.9 (1.55 ~ 2.34)	<0.001
Work hours	1.01 (1.01 ~ 1.01)	<0.001	1.01 (1.01 ~ 1.01)	<0.001	1.01 (1.01 ~ 1.01)	<0.001	1.01 (1.01 ~ 1.01)	<0.001
WSR ≥ 1.205								
WSR	0.74 (0.24 ~ 2.31)	0.604	0.9 (0.59 ~ 1.38)	0.632	0.89 (0.57 ~ 1.37)	0.581	0.8 (0.51 ~ 1.26)	0.339
Work hours	1.01 (1 ~ 1.02)	0.075	1.01 (1 ~ 1.03)	0.076	1.01 (1 ~ 1.02)	0.108	1.01 (1 ~ 1.03)	0.079
Sleep duration (hours)								
< 39	0.99 (0.95 ~ 1.03)	0.512	0.98 (0.95 ~ 1.02)	0.399	0.98 (0.95 ~ 1.02)	0.418	0.99 (0.95 ~ 1.03)	0.569
≥ 39, < 75	0.99 (0.98 ~ 0.99)	<0.001	0.98 (0.98 ~ 0.99)	<0.001	0.99 (0.98 ~ 0.99)	<0.001	0.98 (0.98 ~ 0.99)	<0.001
≥ 75	0.98 (0.9 ~ 1.06)	0.62	0.95 (0.87 ~ 1.04)	0.265	0.95 (0.86 ~ 1.05)	0.336	0.95 (0.83 ~ 1.08)	0.404

Mode I: Age+Gender.

Mode II: Model I + PIR+race+education+marital status.

Mode III: Model II+smoking status+alcohol consumption status+energy+VLTPA+MLTPA+sedentary activity.

Mode IV: Model III+ DM+hypertension+hyperlipidemia+arthritis+HF+CHD+Stroke+COPD+thyroid problem+cancer.

**Figure 3:**
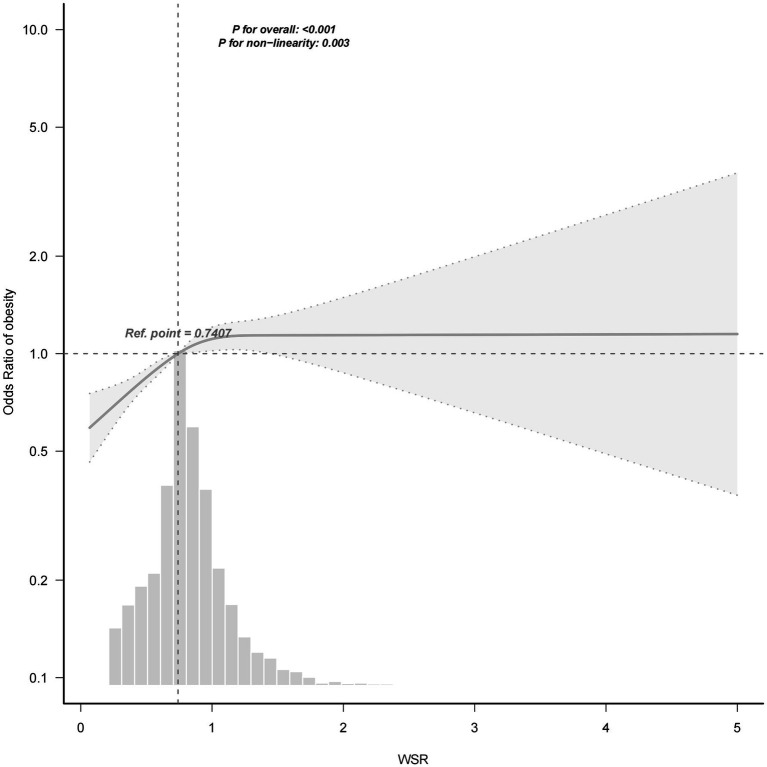
Association between WSR and obesity with RCS. Adjusted for othertime, age, gender, race, marital status, education level, BMI, PIR, alcohol consumption, smoking status, MLTPA, VLTPA, sedentary activity, HF, CHD, DM, hypertension, hyperlipidemia, stroke, COPD, thyroid problem, cancer and arthritis.

**Figure 4:**
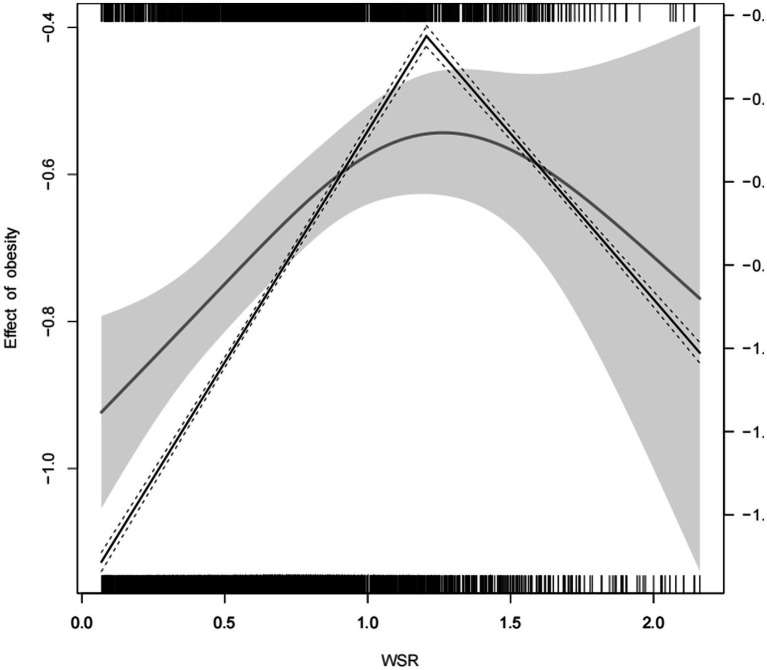
The cutpoint of WSR Adjusted for othertime, age, gender, race, marital status, education level, BMI, PIR, alcohol consumption, smoking status, MLTPA, VLTPA, sedentary activity, HF, CHD, DM, hypertension, hyperlipidemia, stroke, COPD, thyroid problem, cancer and arthritis.

A non-linear relationship was observed between WSR and obesity (Figure 3) with RCS analysis. Table 3 and Figure 4 showed the cutpoint of WSR. The risk of developing obesity increased significantly with WSR < 1.205(OR = 1.9, 95% CI = 1.55–2.34, *p* < 0.001) and no association was observed with WSR ≥ 1.205(OR = 0.8, 95% CI = 0.51–1.26, *p* = 0.339) (Tables 2, 3).

**Table 3:** Threshold effect analysis of WSR on incident obesity.

Outcome	OR (95%CI)	*p* value
One - line linear regression model	1.54 (1.33 ~ 1.77)	<0.001
Two - piecewise linear regression model		
WSR < 1.205	1.9 (1.55 ~ 2.34)	<0.001
WSR ≥ 1.205	0.8 (0.51 ~ 1.26)	0.339
Likelihood Ratio test	–	0.007

Adjusted for age, gender, race, marital status, education level, BMI, PIR, alcohol consumption, smoking status, energy, VLTPA, MLTPA sedentary, HF, CHD, DM, hypertension, hyperlipidemia, stroke, COPD, thyroid problem, cancer and arthritis.

### The correlation between work hours and obesity

Multivariate analysis showed that work hours had positive effect on obesity (OR = 1.01, 95% CI = 1.01–1.01, *p* < 0.001) (Table 2). A linear relationship was identified between work hours and obesity (Supplementary Figure 1).

### The correlation between sleep hours and obesity

Multivariate analysis showed that sleep hours was negative to obesity (OR = 0.99, 95% CI = 0.98–0.99, *p* < 0.001) (Table 2). A linear relationship was identified between sleep hours and obesity (P for non - linearity: 0.061, Supplementary Figure 2). The cutpoints of sleep hours were 39 and 75 (Supplementary Figure 3). When sleep hours are within the range of 39 to 75 h, the risk of developing obesity decreases significantly (Table 2). In the final model, compared with individuals who sleep 39 to 75 h per week, sleep duration < 39 was not significantly associated with obesity (OR = 1.12, 95% CI 1.00–1.26, *p* = 0.056, *p* for trend <0.001) (Table 2). In contrast, sleep duration ≥ 75 was significantly associated with a reduced incidence of obesity (OR = 0.86, 95% CI 0.77–0.97, *p* = 0.011, *p* for trend <0.001) (Table 2). When the sleep duration are less than 39 or more than 75 h, the incidence rate of obesity did not change with the variation of sleep duration in multivariate analysis (Table 2).

## Discussion

In this context, our study represents a pioneering effort to explore the relationship between the WSR and obesity. This is the first article to evaluate the relationship between WSR and obesity. Our research has several key findings: (1) During the period when the value of WSR continues to rise, the incidence of obesity shows an upward trend accordingly. Nevertheless, once the WSR value exceeds the crucial cutoff of 1.205, a saturation effect emerges. (2) A linear correlation was observed between work hours and the incidence of obesity. 3. A “Z” shape was observed between sleep hours and the incidence of obesity. Furthermore, the influence of WSR on obesity was found to be significantly more pronounced than that of work hours or sleep hours considered in isolation. This innovative finding provides an additional dimension for comprehending the intricate associations among work, sleep, and obesity, thereby contributing novel insights to the existing body of literature on the topic.

The interaction between work and sleep has been elaborated in existing research (26, 27). WSR introduces a novel perspective by quantifying the synergistic effect of work and sleep hours in obesity. This approach addresses the limitation of traditional single-behavior analyses (work or sleep hours alone) and fills a long-standing research gap in their interactive effects. Specifically, by normalizing work hours against sleep hours, WSR captures daily time allocation strategies, strategically guiding interventions toward dynamic balance management rather than isolated adjustments to work or sleep. It is worth noting that the impact of WSR (OR = 1.54, 95% CI 1.33–1.77, *p* < 0.001) on obesity is far greater than that of work (OR = 1.01, 95% CI1.01–1.01, *p* < 0.001) or sleep hours (OR = 0.99, 95% CI0.98 ~ 0.99, *p* < 0.001) alone.

Lengthy work hours are generally acknowledged as a major factor contributing to obesity (15, 28) and sleep deprivation (27). Virtanen et al. (29) analyzed 19 cohort studies included 122,078 participants and observed that long work hours (≥ 55 h/week) were associated with the risk of weight gain from normal to overweight (RR = 1.16, 95% CI 1.07–1.26), but not from overweight to obesity (RR = 1.01, 95% CI 0.89–1.14) compared with standard weekly worktime (35–40 h). Another meta-analysis (30) stressed effect of long work hours on obesity with OR = 1.23 (95% CI 1.09–1.39). In our study, we identified a linear relationship between work hours and obesity. As worktime increased, the odds of obesity also rose. The OR was 1.01 (95% CI 1.01–1.01, *p* < 0.001). This result is consistent with existing research findings (14, 30).

Numerous studies have examined closely the relationship between sleep duration and obesity (17–19, 31, 32). A close link between short sleep duration and obesity has been established, but there’s no consensus yet on the connection between long sleep duration and obesity. In our study, there is a negative correlation between sleep hours and obesity (OR = 0.99, 95% CI 0.98–0.99, *p* < 0.001) and the time period of 39 to 75 h makes a major contribution to this relationship (OR = 0.98, 95%CI 0.98–0.99, *p* < 0.001) with the overall data. In the model adjustment, the positive impact of sleep hours < 39 h on obesity was progressively diluted with more variables were incorporated for adjustment compared to individuals with sleep hours 39 to 75 h. In the final model, individuals with sleep hours < 39 h did not show a significant association with obesity (OR = 1.12, 95% CI 1–1.26, *p* = 0.056). However, the proportion of obesity was relatively higher in the group (34.64%, *χ*^2^ = 22.62, *p* < 0.001) and p was less than 0.05 in model I/II/III. Conversely, individuals with sleep hours ≥ 75 h had a lower obesity rate (31.76%, *χ*^2^ = 22.62, *p* < 0.001), with a protective effect against obesity (OR = 0.86, 95% CI 0.77–0.97, *p* = 0.011). Therefore, in our results, the relationship between sleep hours and obesity presents a “Z” shape.

In schoolchildren aged 8 to 17 years, Tambalis et al. (31) observed that 40% of these children had insufficient sleep and insufficient sleep was associated with an increased risk of overweight or obesity (OR = 1.21, 95% CI 1.17–1.25), but the relationship between long sleep duration and obesity was not mentioned. Sa et al. (32) analyzed 1,578 students from U. S. and Korean colleges and observed that obesity was associated with both short (< 7 h/night) (OR = 1.67, 95% CI 1.16–2.41, *p* < 0.01) and long sleep duration (> 9 h/night) (OR = 1.79,95% CI 1.03–3.43, *p* < 0.05), and obesity was related to short sleep among blacks only (*p* < 0.05) in analyses stratified by race and nationality. Jike et al. (33) analyzed 95 articles found that long sleep was significantly associated with obesity (RR = 1.08, 95% CI 1.02–1.15, *p* = 0.010, I^2^ = 0%, *N* = 13). Maugeri et al. (34) analyzed a total of 1,482 participants aged 25 to 65 years and observed that individuals with short sleep duration (<7 h/night) have a 40% increased risk of obesity (OR = 1.40, 95%CI = 1.02–1.94, *p* = 0.047) and long sleep duration (>9 h/night) was associated with lower body weight (*p* = 0.001) and a higher proportion of underweight individuals (*p* = 0.002) compared to individuals with normal sleep duration. A meta-analysis of 6 articles (35) found that short sleep duration increased the risk of incident obesity in both men (OR = 1.26, 95% CI 1.13–1.40) and women (OR 1.36, 95% CI 1.16–1.59), with no significant gender differences. Another meta-analysis (36) analyzed a total of 5,172,710 participants from 153 studies suggests that short sleep not only increase the occurrence of obesity (RR = 1.38, 95%CI 1.25–1.53, *p* < 0.005, I^2^ = 60%, *N* = 16) but is also associated with other health outcomes, such as the mortality outcome, diabetes mellitus, hypertension, cardiovascular diseases, coronary heart diseases. Zheng et al. (37) found that each hour increase in average daily sleep duration was associated with decreased odds of receiving a new diagnosis for morbid obesity (OR = 0.62, 95% CI 0.53–0.73).

The potential mechanisms linking work, sleep, and obesity were elucidated. Dietary alteration is a keystone of weight control. In fact, the actual energy intake is usually 30 to 50% higher than the intake reported by individuals themselves (38). Moreover, the longer the individuals spend working (39), the less able they are to prepare healthy foods on their own. Instead, they frequent restaurants, visit fast-food joints, or rely on food delivery services to fulfill their daily dietary needs, and as a result, the foods they consume also contain less dietary fiber (13, 29, 39, 40). Low level of physical activity is another main factor contributing to obesity (13, 41). The changes in modern work patterns, along with the predominantly office - based work model, have restricted employees’ activity time. Meanwhile, long working hours have left employees mentally and physically exhausted, limiting their time for exercise after work. This is one of the possible mechanisms by which long work hours lead to obesity (13, 42). However, not all researchers think that long work has an effect on physical activity (42). The third possible factor is sleep abnormalities caused by long work hours, including sleep deprivation or sleep disorders (27, 43). Sleep alterations also contribute to the development of obesity to a certain extent (43, 44). The changes in the microecological environment are influenced by work (45) and sleep (46, 47), and the impact of the microecology on obesity is also gradually attracting more attention (48). At the molecular level, the possible mechanisms might be attributed to changes in metabolism-related hormones, such as leptin (43, 49).

In this study, several limitations should be acknowledged. First, the lack of weighted analyses, and the exclusion of individuals who do not work or have no need to work may restrict the generalizability of the results. Second, the cross-sectional nature of the study means that it only examined the potential association between the WSR and obesity, and thus cannot establish a causal relationship. Third, the limited number of covariates included in the analysis may have influenced the interpretation of the results and introduced potential biases. Fourth, this article does not count the staff who are currently looking for jobs. Fifth, the number of working days per week and the type of work have not been mentioned, that may limit the generalizability of results to specific occupational groups. Since the number of working days per week and the daily worktime of the participants were not available, there may be some bias in the analysis.

## Conclusion

Our findings suggest that the WSR, which combines the impact of work and sleep hours, exerts a more substantial impact on the occurrence of obesity compared to work or sleep hours alone. WSR can be used as a new reference indicator for relevant stakeholders to formulate strategies designed to promote employees’ occupational health and global health, thereby reducing the burden of obesity-related diseases.

## Data Availability

The datasets presented in this study can be found in online repositories. The names of the repository/repositories and accession number(s) can be found in the article/Supplementary material.

## Footnotes

^1^ https://www.cdc.gov/nchs/nhanes/about/erb.html

^2^ www.zstats.net
