# Supplementary figures and images for: Positive relationship between Work-to-Sleep hours Ratio and obesity: a cross-sectional study, evidence from NHANES 2017–2023
Source: Front Public Health. 2025 Jun 16;13:1616890. doi: 10.3389/fpubh.2025.1616890 (PMC12206778; doi:10.3389/fpubh.2025.1616890)

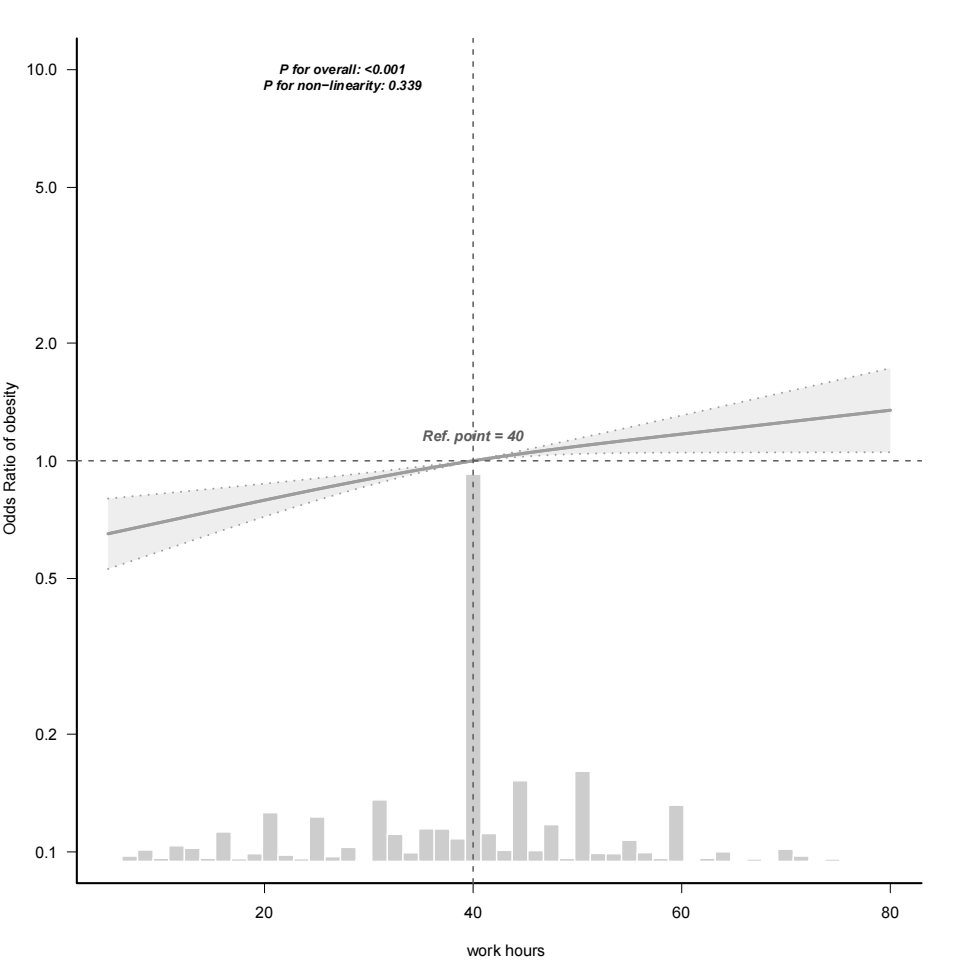

Supplement: Supplementary file 1 [file Image_3.tif]

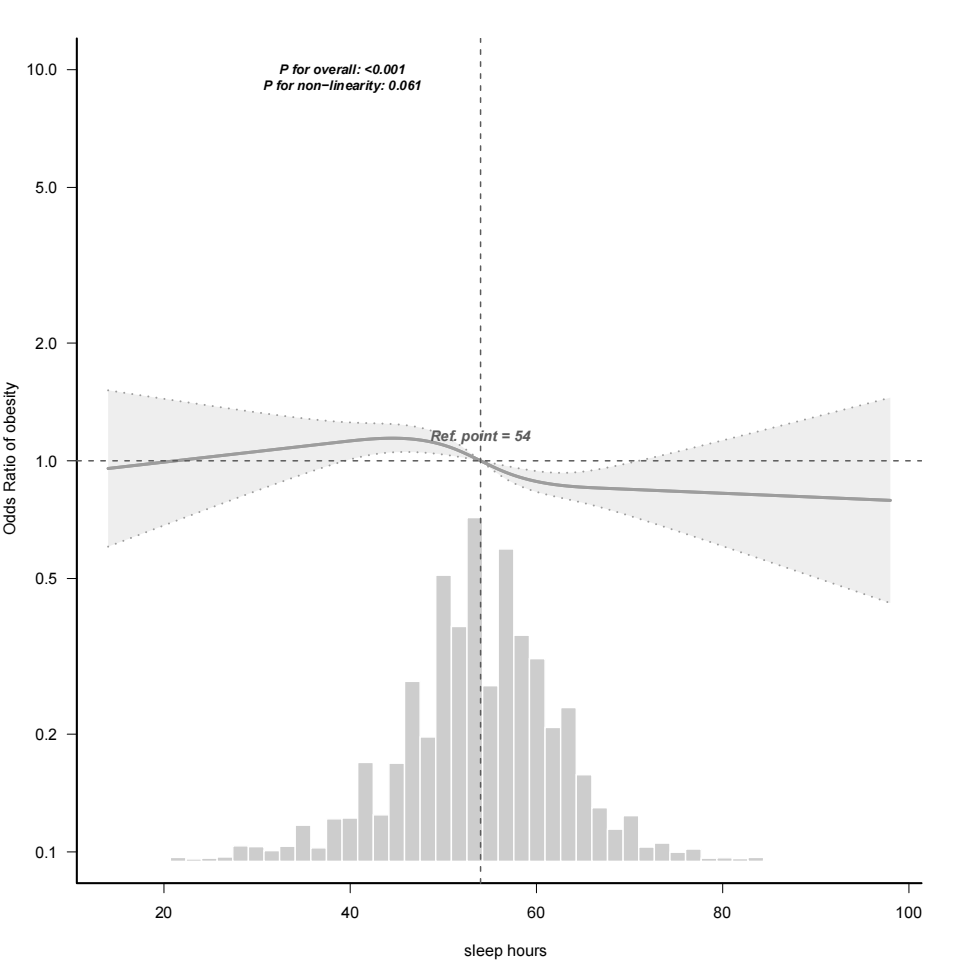

Supplement: Supplementary file 2 [file Image_4.tif]

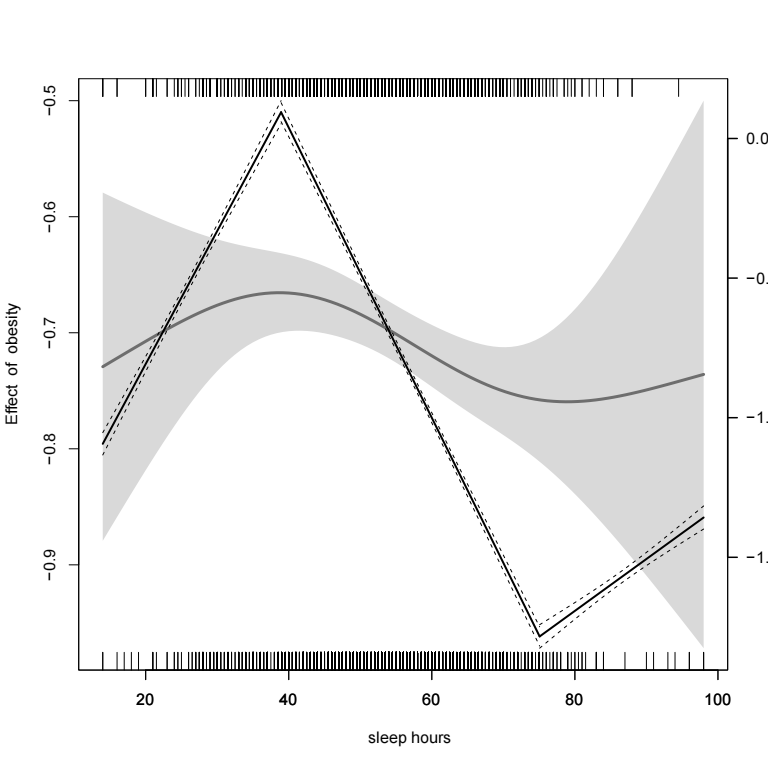

Supplement: Supplementary file 3 [file Image_5.tif]
