# Supplementary material for: Positive relationship between Work-to-Sleep hours Ratio and obesity: a cross-sectional study, evidence from NHANES 2017–2023
Source: Front Public Health. 2025 Jun 16;13:1616890. doi: 10.3389/fpubh.2025.1616890 (PMC12206778; doi:10.3389/fpubh.2025.1616890)
Supplement: Supplementary file 4 [file Table_1.docx]

| Supplementary Table 1 **Univariate regression analysis.** | | | | | |
| --- | --- | --- | --- | --- | --- |
| Variables | β | S.E | Z | *P* | OR (95%CI) |
| Gender |  |  |  |  |  |
| Male |  |  |  |  | 1.00 (Reference) |
| Female | 0.23 | 0.05 | 5.05 | <.001 |  |
| Age ≥ 60 |  |  |  |  |  |
| No |  |  |  |  | 1.00 (Reference) |
| Yes | -0.07 | 0.06 | -1.25 | 0.210 | 0.93 (0.83 ~ 1.04) |
| Education |  |  |  |  |  |
| Below high school |  |  |  |  | 1.00 (Reference) |
| High School or above | 0.02 | 0.07 | 0.30 | 0.763 | 1.02 (0.89 ~ 1.18) |
| PIR |  |  |  |  |  |
| ≥ 1 |  |  |  |  | 1.00 (Reference) |
| < 1 | -0.10 | 0.07 | -1.37 | 0.170 | 0.90 (0.78 ~ 1.04) |
| Marital |  |  |  |  |  |
| No |  |  |  |  | 1.00 (Reference) |
| Yes | 0.13 | 0.05 | 2.40 | 0.016 | 1.13 (1.02 ~ 1.26) |
| Race |  |  |  |  |  |
| Mexican American |  |  |  |  | 1.00 (Reference) |
| Non-Hispanic White | -0.26 | 0.08 | -3.44 | <.001 | 0.77 (0.66 ~ 0.89) |
| Non-Hispanic Black | 0.08 | 0.08 | 1.01 | 0.313 | 1.09 (0.92 ~ 1.28) |
| Other | -0.77 | 0.08 | -9.29 | <.001 | 0.46 (0.39 ~ 0.54) |
| Smoker |  |  |  |  |  |
| Yes |  |  |  |  | 1.00 (Reference) |
| No | 0.18 | 0.06 | 2.75 | 0.006 | 1.19 (1.05 ~ 1.35) |
| Drinker |  |  |  |  |  |
| Yes |  |  |  |  | 1.00 (Reference) |
| No | -0.05 | 0.05 | -0.90 | 0.366 | 0.95 (0.86 ~ 1.06) |
| Work hours | 0.01 | 0.00 | 5.48 | <.001 | 1.01 (1.01 ~ 1.01) |
| Sleep hours | -0.01 | 0.00 | -4.68 | <.001 | 0.99 (0.98 ~ 0.99) |
| WSR | 0.40 | 0.07 | 5.94 | <.001 | 1.49 (1.31 ~ 1.70) |
| Energy | -0.00 | 0.00 | -1.20 | 0.228 | 1.00 (1.00 ~ 1.00) |
| VLTPA | 0.00 | 0.00 | 0.14 | 0.890 | 1.00 (1.00 ~ 1.00) |
| MLTPA | -0.00 | 0.00 | -0.15 | 0.880 | 1.00 (1.00 ~ 1.00) |
| Sedentary | 0.01 | 0.00 | 2.62 | 0.009 | 1.01 (1.01 ~ 1.01) |
| DM |  |  |  |  |  |
| Yes |  |  |  |  | 1.00 (Reference) |
| No | -0.94 | 0.08 | -11.40 | <.001 | 0.39 (0.33 ~ 0.46) |
| Hypertension |  |  |  |  |  |
| Yes |  |  |  |  | 1.00 (Reference) |
| No | -0.87 | 0.05 | -16.70 | <.001 | 0.42 (0.38 ~ 0.46) |
| Hyperlipidemia |  |  |  |  |  |
| Yes |  |  |  |  | 1.00 (Reference) |
| No | -0.42 | 0.05 | -8.30 | <.001 | 0.66 (0.60 ~ 0.73) |
| Arthritis |  |  |  |  |  |
| Yes |  |  |  |  | 1.00 (Reference) |
| No | -0.59 | 0.06 | -10.03 | <.001 | 0.56 (0.50 ~ 0.62) |
| HF |  |  |  |  |  |
| Yes |  |  |  |  | 1.00 (Reference) |
| No | -0.90 | 0.20 | -4.41 | <.001 | 0.41 (0.27 ~ 0.61) |
| CHD |  |  |  |  |  |
| Yes |  |  |  |  | 1.00 (Reference) |
| No | -0.17 | 0.16 | -1.06 | 0.288 | 0.84 (0.61 ~ 1.16) |
| Stroke |  |  |  |  |  |
| Yes |  |  |  |  | 1.00 (Reference) |
| No | -0.30 | 0.18 | -1.68 | 0.092 | 0.74 (0.52 ~ 1.05) |
| COPD |  |  |  |  |  |
| Yes |  |  |  |  | 1.00 (Reference) |
| No | -0.49 | 0.12 | -4.12 | <.001 | 0.61 (0.49 ~ 0.77) |
| Thyroid problem |  |  |  |  |  |
| Yes |  |  |  |  | 1.00 (Reference) |
| No | -0.33 | 0.08 | -4.05 | <.001 | 0.72 (0.61 ~ 0.84) |
| Cancer |  |  |  |  |  |
| Yes |  |  |  |  | 1.00 (Reference) |
| No | -0.04 | 0.09 | -0.45 | 0.650 | 0.96 (0.80 ~ 1.15) |
| OR: Odds Ratio, CI: Confidence Interval | | | | | |
