# Supplementary material for: Positive relationship between Work-to-Sleep hours Ratio and obesity: a cross-sectional study, evidence from NHANES 2017–2023
Source: Front Public Health. 2025 Jun 16;13:1616890. doi: 10.3389/fpubh.2025.1616890 (PMC12206778; doi:10.3389/fpubh.2025.1616890)
Supplement: Supplementary file 5 [file Table_2.docx]

| **Supplementary Table 2 Threshold effect analysis of sleeptime on incident obesity.** | | |
| --- | --- | --- |
| Outcome | OR (95%CI) | P value |
| One - line linear regression model | 0.99 (0.98~0.99) | <0.001 |
| Two - piecewise linear regression model |  |  |
| < 39 | 0.99 (0.95~1.03) | 0.569 |
| ≥ 39, < 75 | 0.98 (0.98~0.99) | <0.001 |
| ≥ 75 | 0.95 (0.83~1.08) | 0.404 |
| Likelihood Ratio test | - | 0.888 |
| Adjusted for age, gender, race, marital status, education level, BMI, PIR, alcohol consumption, smoking status, energy, VLTPA, MLTPA sedentary, HF, CHD, DM, hypertension, hyperlipidemia, stroke, arthritis, COPD, thyroid problem and cancer. | | |
